# Modulation of Serine Metabolism Improves Health and Longevity in Drosophila Melanogaster

**DOI:** 10.1093/geroni/igaf122.4164

**Published:** 2025-12-31

**Authors:** Shengshuai Shan, Ryan Armant, Shelton Swint, Hanna Patel, Kelsey Patterson, Eric Foreman, Jessica Alvarez, Jessica Hoffman

**Affiliations:** Augusta University, Augusta, Georgia, United States; Augusta University, Augusta, Georgia, United States; College of Science and Mathematics, Augusta, Georgia, United States; Augusta University, Augusta, Georgia, United States; Augusta University, Augusta, Georgia, United States; Augusta University, Augusta, Georgia, United States; Augusta University, Augusta, Georgia, United States; Augusta University, Augusta, Georgia, United States

## Abstract

Non-essential amino acids, including L-serine, though often overlooked, are crucial in metabolism and may influence aging. Serine is metabolized through diverse pathways that produce metabolites with different effects on lifespan. Metabolites such as glutathione, spermidine, and glycine are linked to increased longevity, whereas ceramides and sphingolipids are associated with reduced lifespan. These divergent metabolic outcomes may underlie conflicting results in laboratory organisms for previous studies, and the specific metabolites and genes mediating its effects remain largely unidentified. This study aimed to evaluate the effects of serine modulation, via dietary L-serine supplementation and knockdown of key genes involved in L-serine metabolism, on healthspan and lifespan across genetically diverse strains and both sexes of Drosophila melanogaster. Preliminary results indicate that L-serine supplementation significantly extended lifespan and improved healthspan in both sexes. Moreover, knockdown of lace, the first step in sphingolipid biosynthesis and the Drosophila ortholog of serine palmitoyltransferase (SPT), markedly increased lifespan in both sexes, with a more pronounced effect in females. Additionally, knockdown of serine hydroxymethyltransferase (SHMT), which bidirectionally converts glycine to serine, significantly increased lifespan in both sexes. Genetic manipulations increased longevity to a greater degree than serine supplementation. These findings suggest that metabolic reprogramming, achieved by targeting specific branches of the serine metabolic pathway, may be a key mediator of health outcomes. Future directions will analyze metabolomic profiles of serine supplemented and knockdown flies for insights into the molecular mechanism that drive their effects. In conclusion, our results highlight serine metabolism as a potential pro-longevity target for intervention studies.

